# Brazilian Green Propolis: Anti-Inflammatory Property by an Immunomodulatory Activity

**DOI:** 10.1155/2012/157652

**Published:** 2012-12-19

**Authors:** Joleen Lopes Machado, Anne Karine Martins Assunção, Mayara Cristina Pinto da Silva, Aramys Silva dos Reis, Graciomar Conceição Costa, Diêgo de Sousa Arruda, Bruno Alves Rocha, Mirela Mara de Oliveira Lima Leite Vaz, Antonio Marcus de Andrade Paes, Rosane Nassar Meireles Guerra, Andresa Aparecida Berretta, Flávia Raquel Fernandes do Nascimento

**Affiliations:** ^1^Department of Pathology, Laboratory of Immunophysiology, Biological and Health Sciences Center, Federal University of Maranhão (UFMA), 65085-580 São Luis, Brazil; ^2^Apis Flora Industrial e Comercial Ltda, 14020-670 Ribeirão Preto, SP, Brazil; ^3^Department of Science Physiology, Laboratory for Teaching and Research of Physiology, Federal University of Maranhão (UFMA), 65085-580 São Luis, Brazil

## Abstract

The immunomodulatory and anti-inflammatory activities of green propolis extracts from *Apis mellifera* were investigated using acute and chronic inflammation models. Swiss mice were anesthetized and a cotton pellet granuloma was implanted in subcutaneous tissue. Then the mice were divided into six groups and received apyrogenic water or different propolis extracts by oral route (5 mg/kg). According to the treatment the groups were designated as E1A, E1B, E10, E11, and E12. The control group received apyrogenic water. The treatment was performed by six days when the mice were killed. The blood and the bronchoalveolar lavage (BAL) were collected to measure the leukocyte recruitment. In acute pulmonary inflammation, Balb/c mice received lipopolysaccharide (LPS) of *Escherichia coli* by intranasal route for three days. Concomitantly the mice received by oral route apyrogenic water (control) or E10 and E11 propolis extracts. BAL was performed to assess the inflammatory infiltrate and cytokine quantification. The results showed that the E11 extract has anti-inflammatory property in both models by the inhibition of proinflammatory cytokines and increase of anti-inflammatory cytokines suggesting an immunomodulatory activity.

## 1. Introduction

Green propolis is well known due to the color [1, 2] and is produced by *Apis mellifera* honeybees that utilize *Baccharis dracunculifolia* DC (Asteraceae), a common species found in the Brazilian cerrado, as the main plant source [3]. Several studies have reported green propolis to have antiulcerogenic [3], anti-inflammatory [4], antimutagenic [5], antifungal [6–8], immunomodulatory [9], angiogenesis [10], and antioxidant [11] properties. The biological activities of propolis are due to its high levels of phenolic acids [12], while flavonoids are considered responsible for the activities of the European propolis extracts [13]. The typical constituents of Brazilian green propolis are caffeoylquinic acid and prenylated derivatives of cinnamic acid, such as artepillin C and baccarin [14, 15].

The immunomodulatory effects of natural substances have been considered as alternative adjuvant therapies in the treatment of various diseases [16]. In the case of propolis, this effect has been associated with a combination of different constituents [17]. The administration of green propolis in animals subjected to chronic stress increased the generation of hydrogen peroxide, suggesting that this product modulated the activation of macrophages [18]. In an *in vivo* model of chronic inflammation, it has been also demonstrated that green propolis extract suppresses cell migration without compromising collagen deposition. Thus, green propolis may be used to control the inflammatory response [19]. Due to the previous knowledge of the possible effect of the green propolis on the inflammatory immune response, this study investigated the local and systemic effect of different extracts of Brazilian green propolis on the inflammatory response in different experimental models. 

## 2. Material and Methods

### 2.1. Collection and Preparation of Propolis Extracts

This study used lyophilized samples of aqueous extracts of propolis produced by Apis Flora, Ribeirão Preto, SP, Brazil. The extracts production follows a patented and standardized process (PI 0405483-0), published in the Revista de Propriedade no. 1778 of 12.01.2005 [20]. In this process, different propolis samples from Minas Gerais (MG), São Paulo (SP), Rio Grande do Sul (RS), Paraná (PR), and Santa Catarina (SC) were used. Extracts were mixed in standardized concentrations, giving rise to a pool of samples, where green propolis was predominant. The lyophilized extracts were numbered according to the type of extraction: E1A and E1B extracts were prepared from the direct extraction of propolis (pool) using industrialized solvents, while E10, E11, and E12 extracts were obtained from the concentration and alkaline hydrolyze, according to de Andrade et al. [21] with modifications, of the standardized propolis extract (EPP-AF) and the solubility in purified water.

### 2.2. Chemical Characterization of Propolis Extracts by HPLC

Quantitative analysis of the propolis extracts was carried out in a high-performance liquid chromatography (HPLC-Shimadzu) equipped with a CBM-20A controller, an LC-20AT quaternary pump, an SPD-20A M diode array detector, and a Shimadzu LC software, version 1.21 SP1. A Shim-pack CLC-ODS (M) (4.6 mm × 250 mm, particle diameter of 5 mm, pore size 100 Å) Shimadzu column. The mobile phase consisted of a gradient of methanol (JT Baker) and acidified water with formic acid (0.1% v/v) ranging from 20% to 95%. A run of 77 minutes at a flow rate of 0.8 mL/min, with detection at 275 nm, was performed. The following compounds were used as standards in the HPLC analysis: caffeic acid (Fluka), p-coumaric acid (Fluka) and trans-cinnamic acid (Fluka), artepillin C (Wako), gallic acid (Synth), isosakuranetin (ChromaDex), and 4′O-methyl-ether aromadendrin. These compounds were previously isolated and identified as described by [22] and kindly provided by the authors. The water was treated in Milli-Q purification system. The lyophilized propolis samples (*n* = 3) named as E1A, E1B, E10, E11, and E12 were diluted in methanol/water and homogenized using an ultrasonic bath. After filtration with a 0.45 *μ*m filter, 15 *μ*L of each sample was injected into the HPLC system.

### 2.3. Animals

Swiss and Balb/c mice (8–12 weeks, 25–30 g) were used. Animals were assigned by the mouse breeding facilities of Federal University of Maranhão. The animals received water and food *ad libitum*, while being maintained and handled in accordance with the rules of SBCAL (Brazilian Society of Animal Science Lab) Protocol (CEP/UEMA no. 010/2007).

### 2.4. Cotton Pellet Granuloma

**Figure 1:**
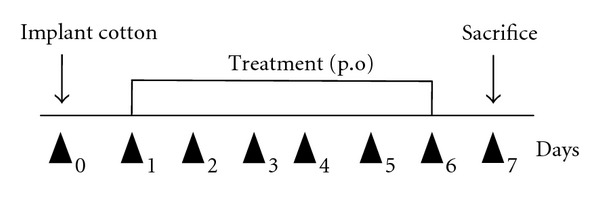
Treatment protocol for the granuloma model.

The method adopted for granuloma formation was described by Swingle and Shideman [23], and it was adapted in our laboratory. Animals were divided into six groups (1 control—5 experiments) (*n* = 6/group). The control group received 200 *μ*L of apyrogenic water orally (p.o) (Figure 1). The experimental groups received different propolis extracts at a dose of 5 mg/kg p.o for six days and in accordance with the received extract were termed E1A, E1B, E10, E11, and E12. The animals were anesthetized via an intramuscular injection with a solution of 2% xylazine chloridate (20 mg/kg) and 5% ketamine chloridate (25 mg/kg) in a 2 : 1 ratio. A small incision was made in the skin of the dorsal region to introduce a subcutaneous implant of sterilized cotton (9 mg—prior to introduction). The animals were sacrificed on day 7 after implantation, when the cotton implants were removed and weighed to obtain wet weight (total weight). Then, the implants were lightly pressed into sheets for the subsequent differential counting of cells found in the granuloma after staining the slides with an Instant-Prov Kit (Newprov, Pinhais, Brazil) as described by the manufacturer. Subsequently, the cotton implants were dried in a stove at 37°C for 48 hours and weighed to measure the granuloma dry weight, which corresponded to the cell weight formed in the granuloma. From the final weight, the present edema in the granuloma was calculated using the following formula: weight of edema present in the granuloma = Ps – Pi, where Pi is the initial weight (total) and Ps is the dry weight.

#### 2.4.1. Evaluation of Hematological Parameters

To determine the hematological parameters, 100 *μ*L of blood was collected from the mice seven days after cotton implantation. Blood was stored in 1.5 mL tubes with ethylenediaminetetraacetic acid (EDTA) as anticoagulant. An automated hematology analyzer (Poch-100iV Diff, Sysmex Corp) was used. The following parameters were analyzed: red blood cells, hemoglobin, hematocrit, mean corpuscular volume (MCV), mean corpuscular hemoglobin (MCH), mean corpuscular hemoglobin concentration (MCHC), red-cell distribution width (RDW), and number of leukocytes, neutrophils, lymphocytes, and platelets.

#### 2.4.2. Quantification of the Number of Cells in Lymphoid Organs

After the animals were sacrificed, the spleen was removed, weighed, and triturated in 5 mL of phosphate-buffered solution (PBS) using a sieve. To obtain the bone marrow cells, the femur was removed and perfused with 1 mL of PBS. The inguinal lymph nodes were removed, weighed and triturated in 1 mL of PBS. The cell suspensions were kept in an ice bath. All cell counts were performed using crystal violet solution (0.05% in 30% acetic acid) as described by Maciel et al. [24].

#### 2.4.3. Bronchoalveolar Lavage

The trachea of the animals was exposed, fitted with a cannula, and 1 mL of cold PBS was injected in the bronchoalveolar space with a syringe. After a short massage on the chest, the solution was aspirated at least three times. The lungs were collected, weighed, and fixed in 10% formalin for subsequent histopathology analysis. To determine the total number of cells in the bronchoalveolar lavage, cell suspensions were stained with crystal violet (0.05%) in 30% acetic acid at a ratio of 9 : 1. Cells were counted in a Neubauer chamber using an optical microscope (×400). For differential counting slides were prepared using a citospin (800 rpm/3 min) and then were fixed and stained using an Instant-Prov Kit (Newprov, Pinhais, Brazil) [25].

### 2.5. Induction of Acute Pulmonary Inflammation by Instillation of Lipopolysaccharide (LPS)

**Figure 2:**
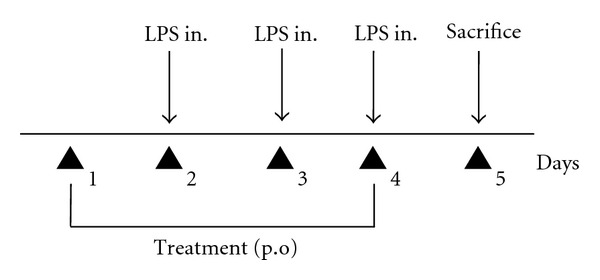
Treatment protocol for the LPS induced pulmonary inflammation model.

Balb/c mice were divided into three groups (*n* = 5/group): control, E10 and E11. The control group received 200 *μ*L of apyrogenic water, and the groups were designated as E10, and E11 according to the extract received in a dose of 5 mg/kg, p.o. The animals were anesthetized intramuscularly with 0.4 mL of 2% xylazine chloridrate (20 mg/kg) and 5% ketamine chloridate (25 mg/kg). With a micropipette (Gilson), 10 *μ*L of LPS solution (1 mg/mL sterile PBS) was given by the aerogenic route using nasal instillation. This induction was done on three consecutive days (Figure 2). One day before the first LPS application, the control, E10, and E11 treatment was started. This treatment continued for four days, and the sacrifice of the animals was performed 24 hours after the last application of LPS, when the bronchoalveolar lavage was performed as mentioned above [26].

#### 2.5.1. Cytokine Determination

The concentration of the IL-6, IL-10, TNF-*α*, and TGF-*β* cytokines was measured in the bronchoalveolar lavage taken from the animals using the sandwich ELISA method, according to the manufacturer's specifications (eBioscience, San Diego, CA USA). Captured monoclonal antibodies for each cytokine were incubated overnight at 4°C in Corning Costar 9018 plates (100 *μ*L/well). After incubation the plates were washed with 0.05% PBS+Tween 20, and nonspecific reactions were blocked by the addition of 10% fetal calf serum (FCS) (200 *μ*L/well) for 60 min. The plates were washed and samples added (100 *μ*L/well). After 24-hour incubation at 4°C, plates were washed and the detection antibody added (100 uL/well). The plates were incubated for 1 hour at room temperature. After further washing, conjugated avidin peroxidase was added, and the plates were incubated for 30 min at room temperature. The colorimetric reaction was performed by adding 100 *μ*L of the TMB substrate per well. Then the blocking reaction was carried out by the addition of 50 *μ*L/well of 2N H_2_SO_4_, and the absorbance was measured at 450 nm. Optical densities (OD) values were converted to pg/mL or ng/mL based on the curves obtained with different concentrations of recombinant cytokines.

### 2.6. Statistical Analysis

Unpaired Student *t*-tests were performed, adopting *P* < 0.05 as a significant value. To compare the propolis extracts, we conducted further analysis of the six standards separately; the results were analyzed by a *one-way* ANOVA followed by Bonferroni's multiple comparisons. Statistical analysis was performed using the GraphPad Prism Software (5.0), and, for further calculations, the Microsoft Excel 2010 program was used.

## 3. Results

### 3.1. Chemical Characterization of Aqueous Extracts of Green Propolis

The predominant standard in all the tested aqueous extracts of green propolis was p-coumaric acid, but all other standards (caffeic acid, cinnamic, aromadendrin, and isosakuranetin) were also detected in all extracts at a lower level. Artepillin C was not detected in the 1A extract only. The statistical differences in the concentration of these compounds are shown in Table 1.

### 3.2. The Effect of Propolis Extracts Treatment on the Cotton Pellet Granuloma

**Figure 3:**
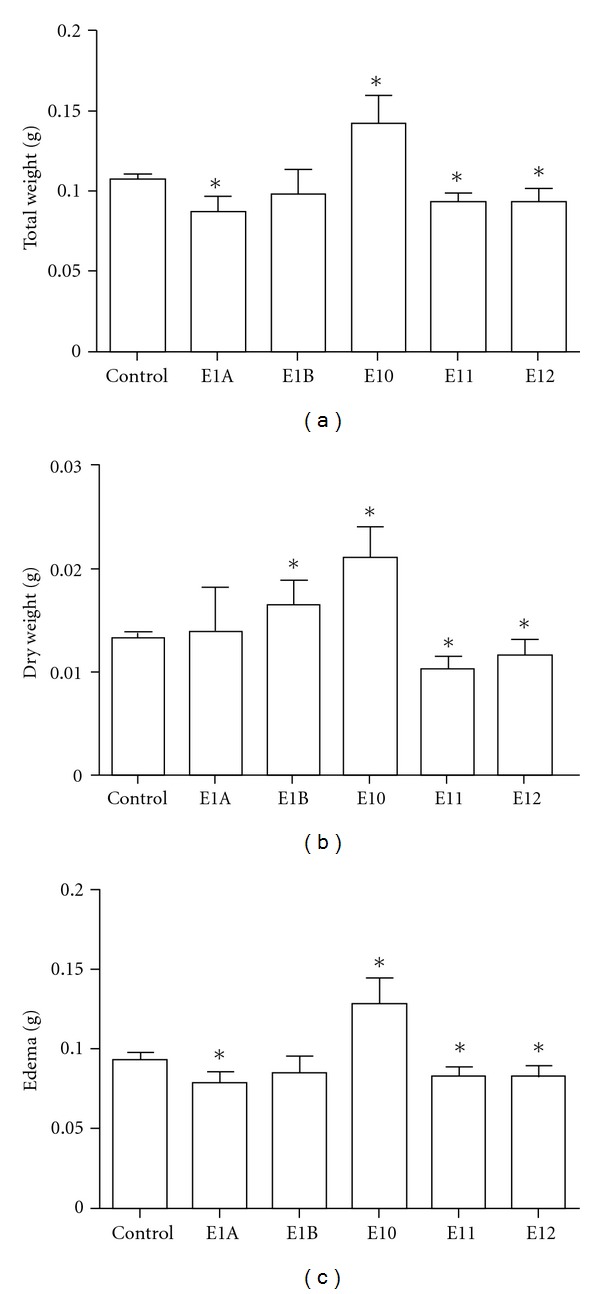
The effect of treatment with an aqueous propolis extract from *Apis mellifera* in cotton pellet granuloma. Swiss mice that received a cotton implant on the back were treated orally for 6 days with a daily dose of 5 mg/kg and were compared to controls, which received apyrogen water at the same intervals. At the end of treatment, the cotton implants were removed, and total wet weight (a) and the dry weight (b) were determined after 48 hours at 37°C. The difference between wet weight and dry weight determined the edema (c). The data represent the mean ± SD of six animals/group. *P < 0.05 compared to control group.

Several types of the tested extracts induced different effects in the formation of granuloma, both in relation to total weight (Figure 3(a)), dry weight (Figure 3(b)) and edema (Figure 3(c)). E1A induced a decrease in the total weight of the granuloma, and edema when compared to the control, while E1B did not induce any changes. E10 induce a proinflammatory effect, while E11 and E12 exhibited anti-inflammatory effect, in the 3 total weights.

#### 3.2.1. The Effect of Propolis Extracts Treatment on the Hematological Parameters of Animals with Cotton Pellet Granuloma

Extracts E1A, E1B and E12 did not induce hematological changes in animals. However, E10 and E11 induced an increase in the number of leukocytes and other white blood cells when compared to the control group, while E11 also induced a reduction in platelet count compared to the control group (Table 2).

#### 3.2.2. The Effect of Treatment with Propolis Extracts on the Cellularity of the Lymphoid Organs of Animals with Induced Cotton Pellet Granuloma

Extracts E1B, E10, E11, and E12 induced a decrease in the number of marrow cells while extracts E1A, E1B, and E10 induced a decrease in the number of spleen cells, when compared to the control group. Conversely, extracts E1A, E10, and E11 induced an increase in the number of lymph node cells compared to the control group (Table 3).

#### 3.2.3. The Effect of Propolis Extracts Treatment on Pulmonary Inflammation Induced by Subcutaneous Implantation of Cotton

**Figure 4:**
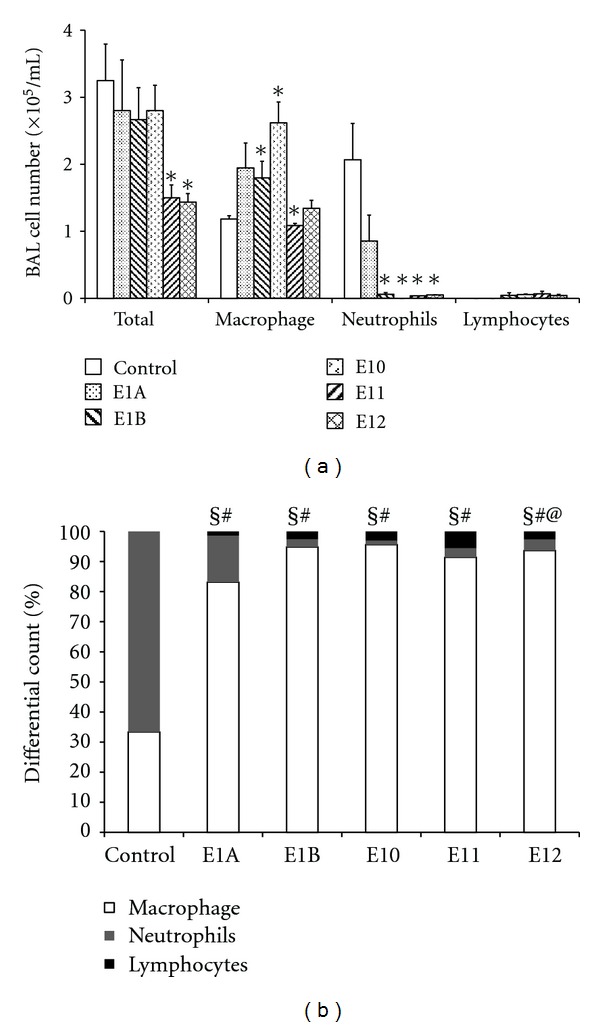
The cellular profile of the bronchoalveolar lavage fluid of mice with granulomatous inflammation. Swiss mice that were treated orally for six days with a daily dose of 5 mg/kg were compared to controls, which received apyrogenic water at the same intervals. After treatment, BALs were collected and the different cell-types were counted. (a) The number of cells in the bronchoalveolar lavage fluid of mice with granulomatous inflammation orally treated with extracts of propolis from *Apis mellifera*. (b) The percentage of cells in bronchoalveolar lavage fluid of mice with granulomatous inflammation orally treated with extracts of propolis from *Apis mellifera*. The data represent the mean ± SD of six animals/group.  *P < 0.05 compared to control group. ^§^Monocytes, *P* < 0.05 compared to control. ^#^neutrophils, *P* < 0.05 compared to control. ^@^lymphocytes, *P* < 0.05 compared to control.

To evaluate whether the effect of the treatment with propolis extracts was due to systemic inflammation of the lungs, pulmonary inflammation in animals with granuloma was evaluated. A decrease in the number of inflammatory cells within the total BAL cell count was observed after treatment with the extracts E11 and E12. A significant increase in the number of macrophages was observed in the BAL collected from animals treated with E1B and E10, while the number of neutrophils significantly decreased following treatment with extracts E1B, E10, E11, and E12, when compared to control group. The number of lymphocytes did not change (Figure 4(a)). In all treated groups the predominant cell types within the BAL were macrophages and neutrophils, except for the group that was treated with E12, where lymphocytes were the predominant cell type (Figure 4(b)).

### 3.3. The Effect of Propolis Extracts Treatment on LPS-Induced Pulmonary Inflammation

**Figure 5:**
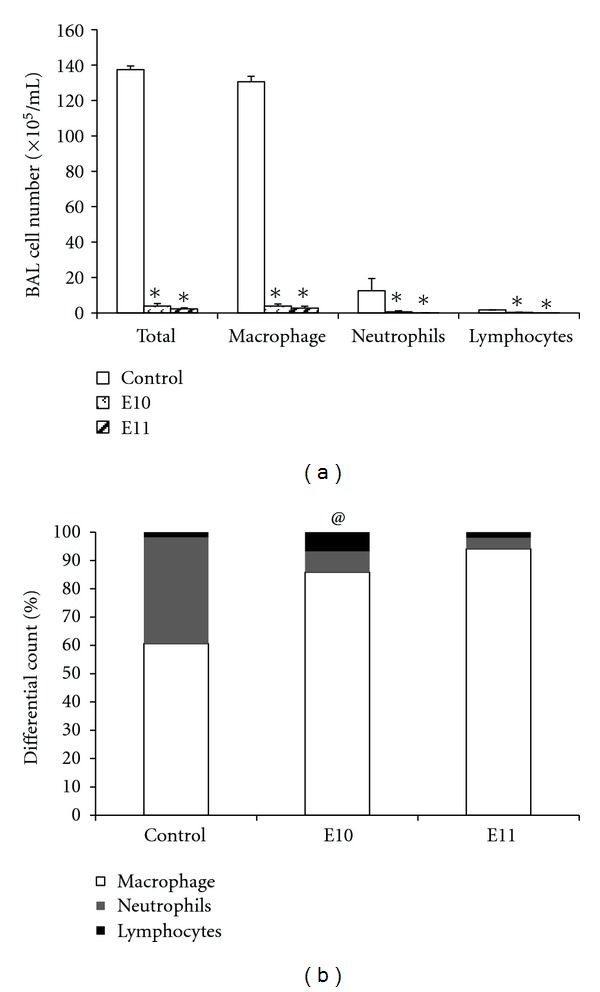
The cellular profile of bronchoalveolar lavage fluid of mice with lung inflammation induced by LPS. Balb/c mice that were treated orally for 4 days with a daily dose of 5 mg/kg were compared to controls, which received apyrogen water at the same intervals. Induction was done for three consecutive days. One day before the induction of inflammation by LPS, animals were treated with apyrogen water (control), maintained for four days, and then the animals were sacrificed 24 hours after the last LPS treatment, when the bronchoalveolar lavage was performed. (a) The number of cells in the bronchoalveolar lavage fluid of mice with pulmonary inflammation induced by LPS intranasally (in.). (b) The percentage of cells in the bronchoalveolar lavage fluid of mice with pulmonary inflammation induced by LPS intranasally (in.). The data represent the mean ± SD of six animals/group.  *P < 0.05 compared to control group treated with propolis extracts from *Apis mellifera*. ^@^lymphocytes, *P* < 0.05 compared to control.

Given the effects observed in animals with pulmonary inflammation granuloma, the impact of propolis on acute pulmonary inflammation, induced by LPS, was assessed. For this test, we selected the extracts E10 that induced a proinflammatory effect and E11 that induced an anti-inflammatory effect in the granuloma model. Treatment with E10 and E11 induced a decrease in the number of inflammatory cells, macrophages, neutrophils, and lymphocytes in the BAL (Figure 5(a)) while lymphocyte predominance was only observed in the E11-treated group (Figure 5(b)).

#### 3.3.1. The Effect of Propolis Extracts Treatment on Cytokine Production in the Supernatant of the Bronchoalveolar Lavage of Animals Treated with LPS-Induced Pulmonary Inflammation

**Figure 6:**
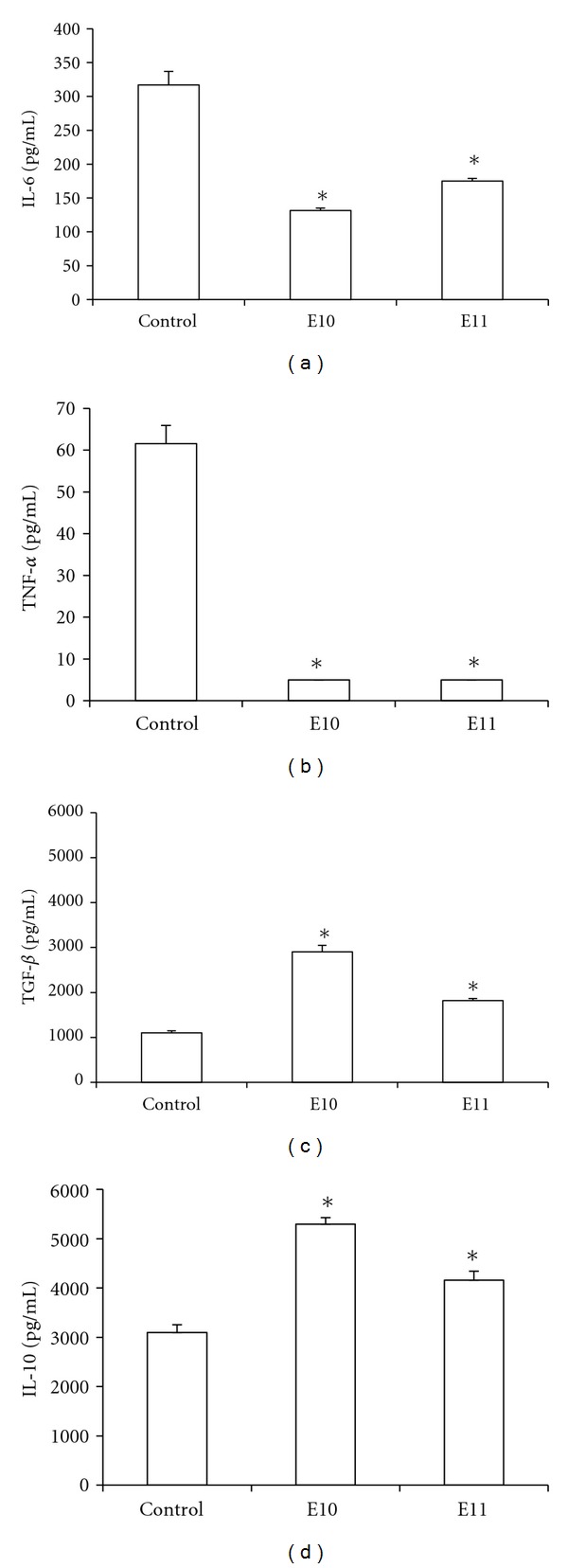
The effect of treatment with propolis extracts of *Apis mellifera* on cytokine production in the supernatant of bronchoalveolar lavage. Balb/c mice that were treated orally for 4 days with a daily dose of 5 mg/kg were compared to controls which received apyrogenic water at the same intervals. Induction was done for three consecutive days. One day before the induction of inflammation by LPS, the animals were treated with apyrogen water (control) for four days, and then the animals were sacrificed 24 hours after the last LPS treatment when the bronchoalveolar lavage was performed. The concentrations of IL-6 (a), TNF-*α* (b), TGF-*β* (c), and IL-10 (d) were determined by ELISA. The data represent the mean ± SD of six animals/group.  *P < 0.05 compared to control group.

There was a decrease in the concentration of TNF-*α* and IL-6 in the groups treated with E10 and E11 when compared to the control. On the other hand, there was an increase in TGF-*β* and IL-10 in the both groups when compared to control group (Figure 6).

## 4. Discussion

This study evaluated the effect of aqueous extract of green propolis in two different models of inflammation. The therapeutic activities of aqueous extracts of propolis are rarely investigated despite of its potential antioxidant and anti-inflammatory activity, and better absorption than ethanolic extract [27]. It has been shown here that the aqueous extract of propolis has a strong anti-inflammatory potential, in pulmonary and granulomatous model, particularly in the first case. The model for induced cotton pellet granuloma used in this study is a method that has been widely used to evaluate the transudative, exudative, and proliferative components of inflammatory diseases since the wet and dry weights of the cotton implant allow the inference of the edema and inflammatory infiltration [23]. 

This model was used to evaluate the effect of oral treatment with aqueous extracts of propolis. After six days of treatment the E10 extract was observed to induce an increase in edema and inflammatory infiltrate (Figure 3) suggesting a proedematogenic and proinflammatory effect. Moreover, the E11 extract induced the opposite effect since it reduced edema and cell infiltration. The E11 and E12 extracts decreased both total weight (Figure 3(a)), as the dry weight, indicating the inflammatory infiltrate (Figure 3(b)) and edema (Figure 3(c)) acting as anti-inflammatories. The E1A and E1B extracts induced variable effects, and the E1A extract induced a decrease in the total weight (Figure 3(a)) and edema (Figure 3(c)), whereas the E1B extract did not affect the total weight of the cell (Figure 3(a)) or edema (Figure 3(c)).

The differences observed between the effects of the extracts are probably due to the chemical characteristics of each extract. All extracts contained caffeic acid, p-coumaric and cinnamic, aromadendrin and isosakuranetin. Artepillin C was found in all extracts except for E1A. However, there was a difference in the concentration of these compounds between extracts (Table 1). Taking the extracts E10 and E11, which showed difference in the granuloma model, as an example, extract E10 showed a higher concentration of all the markers except for p-coumaric acid and aromandendrin when compared to the extract E11. Additionally, the marker with the highest concentration in E10 was artepeillin C, whereas for E11 it was p-coumaric acid (Table 1).

The action of acids (p-coumaric, cinnamic and caffeic) in the granuloma model was tested at a dose of 1 mg/kg, but no significant difference in any of the parameters evaluated was observed (data not shown), which makes us think that the anti-inflammatory effect observed in this study is not due to the action of these phenolic acids alone, but by an additive effect between them. However, in other experimental models other actions of these phenolic acids have been demonstrated, such as cinnamic acid isolated from propolis that was shown to act upon both innate and acquired immunity, stimulating the proliferation of lymphocytes, and inducing the production of cytokines [28]. It has also been demonstrated that caffeic acid may be useful in controlling the growth of tumors in experimental models [29]. Barros et al. [30] utilized the gastric ulcer model to demonstrate that phenolic acids have antiulcerogenic activity. Moreover, *in vivo* studies with artepelin C, the main component of green propolis, showed the inhibition of prostaglandin E2 (PGE2) production in a model of peritonitis [31], demonstrating its effect on inflammation. Moura et al. [10] showed that the antiangiogenic and anti-inflammatory activity in aqueous extracts of green propolis seems to be due to the presence of artepillin C, caffeoylquinic acid and CAPE in the extract.

The complex chemical composition of propolis may be the answer to the existence of numerous activities related to this Beekeeping product. Phenolic compounds are among the most prominent components of propolis because they are considered responsible for most of its properties. This is due to the fact that phenolic compounds exert multiple effects, such as antioxidant, antitumor, anti-inflammatory, and anticancer, among other effects [32–34]. Furthermore, the interaction between them, in an extract containing different concentrations, may result in the different effects observed.

Considering the differences observed between the E10 and E11 extracts, such as the biological activity in the granuloma model, as compared to the observed chemical markers, we investigated whether the extracts had any differential action in a systemic way. Firstly hematological data was evaluated, as this provides important indicators of physiological and pathological changes in humans and animals [35]. This data showed an increase in the number of leukocytes in the animals treated with E10 and E11 extracts (Table 2). Those leukocytes were mainly lymphocytes and neutrophils. The results further demonstrated that the E10 and E11 extracts, beyond altering cell migration to the formation of granulomas, were also interfering in the recruitment of cells from the marrow into the blood and from this to the tissues.

To clarify whether the changes observed in the blood would be due to changes in cell production in the bone marrow and to check whether there were changes in recruitment and/or proliferation of leukocytes to the lymph node and spleen, the cells of these organs were also quantified. There was a decrease in the number of cells in the marrow of animals treated with the E1B, E10, E11, and E12 extracts, which may suggest an increase in cell recruitment from the marrow to the blood and explain the increased number of leukocytes in blood. We also observed a decrease in spleen cell in the E1A, E1B, and E10 extracts and increased inguinal lymph node cells by E1A, E10, and E11 extracts (Table 3).

As the E10 and E11 extracts showed systemic effects, we evaluated the effect of the treatment on pulmonary inflammation induced by granuloma. For this, we used the total cell count and differentials in BAL, as this is the standard indicative of inflammatory response in the respiratory tract, where pulmonary macrophages are the predominant cells (>90%) in healthy animals [36].

We observed a significant decrease in the inflammatory infiltrate in the animals treated with E11 and E12 extracts. In the differential count an increase in the number of macrophages induced by extract E10 and a decrease induced by E11 extract were observed (Figure 5(a)). These results suggest that the E10 and E11 extracts have the ability to modulate cell recruitment in the inflammatory area, reducing neutrophil inflammation which could be harmful to the inflamed tissue [36].

From the obtained results that showed that the E10 and E11 extracts induced opposite effects in both the model of granuloma as well as in the macrophage infiltration in the BAL, we investigate whether these opposing effects would also be observed in pulmonary inflammation induced by LPS, a widely used proinflammatory agent. However, in this model, both extracts showed anti-inflammatory effects, as a decrease in the total number of inflammatory cells, macrophages and neutrophils were observed (Figure 5(a)). Moreover, when the percentage of lymphocytes was quantified, it was noted that both extracts induced an increase in the recruitment of these cells (Figure 5(b)). These results strongly suggest that the E10 and E11 extracts modulate cellular responses in the model of LPS-induced pulmonary inflammation via changing the profile of immune cells involved in this process.

Given that all the inflammatory process is conducted with the involvement of cells and their mediators in which the cytokines [37], in particular those produced by variations in T lymphocytes patterns, can orchestrate an immune response in accordance with the differential *milieu* of cytokines produced [38]. We therefore investigated whether the observed inhibition of pulmonary inflammation was related to modulation of proinflammatory cytokines such as IL-6 and TNF-*α* and cytokines such as IL-10 and TGF-*β* in the lung. In fact, we confirmed that the E10 and E11 extracts induced a reduction in the secretion of IL-6 and TNF-*α* and an increase in TGF-*β* and IL-10, which may explain the inhibition of inflammation observed (Figure 6), in particular when taken into account that, in a normal lung, TGF-*β* is involved in maintaining lung homeostasis by restricting the pathological inflammatory responses [39].

The cytokine IL-6, in addition to being one of the most studied cytokines, has pleiotropic action that influences the antigen-specific immune responses and inflammatory reactions [40] self-limiting inflammatory response [41], contributing along with the profile presented by other cytokines studied in the resolution of the inflammatory response induced by LPS. Our results corroborate that of Khayyal et al. [42], who showed that the use of aqueous extracts of propolis can reduce nocturnal attacks of asthma, which were associated with a decrease in proinflammatory cytokines (TNF-*α*, IL-6, IL-8) and an increase in IL-10. Likewise, Sy et al. [43] used a model of pulmonary inflammation induced by ovalbumin (OVA) and demonstrated that treatment with propolis inhibits pulmonary inflammation and decreases serum levels of IgE and IgG1.

However, our results disagree with those obtained by Orsatti et al. [9], who showed that the administration of ethanol extract of Brazilian green propolis at a dose of 200 mg/kg for 3 consecutive days in mice increases both the innate immunity and also the expression of proinflammatory cytokines (IL-1 and IL-6). However, it is necessary to emphasize that Orsatti et al. [9] used a greater dose than that used in our work, as well as having used an ethanol extract. In our model it was observed that one which induced an increase of TGF-*β* and IL-10, which are regulatory cytokines that contribute to the regulation of the inflammatory process [44, 45] by adding it to the modulating effect of proinflammatory cytokines. Our data may suggest that the E10 and E11 extracts demonstrated local and systemic anti-inflammatory action resulting from an immunomodulatory action.

These effects may be due to synergic effect and/or additive effect of various green propolis compounds thereby decreasing the inflammation observed. The extracts are also capable of modulating the production of proinflammatory and anti-inflammatory cytokines, preventing amplification of the inflammatory process in the pulmonary site. Thus, the tested extracts may become a new therapeutic alternative for use in allergic diseases and inflammation in the respiratory tract.

Further studies will be conducted to characterize the bioactive constituents in other models of inflammation and to evaluate the antioxidant potential of these extracts *in vivo* since the phenolic compounds are found in large quantities in green propolis and are able to interfere with inflammatory processes, therefore investigating the effect of bee products on the immune system.

## Figures and Tables

**Table 1:** Chemical characterization of aqueous extracts of green propolis (mg/g).

Markers	Samples				
	E1A	E1B	E10	E11	E12
Caffeic acid	7.33 ± 0.04a	1.64 ± 0.02b	3.49 ± 0.11c	2.83 ± 0.03d	3.24 ± 0.05e
p-Coumaric acid	37.71 ± 0.33a	10.25 ± 0.04b	9.43 ± 0.30c	12.46 ± 0.10d	19.57 ± 0.18e
Cinnamic acid	1.19 ± 0.04a	0.42 ± 0.02b	0.53 ± 0.02c	0.46 ± 0.01bc	0.80 ± 0.04d
Aromadendrin	4.62 ± 0.20a	0.80 ± 0.08bc	0.56 ± 0.02b	0.88 ± 0.11c	1.44 ± 0.18d
Isosakuranetin	16.30 ± 0.26a	9.51 ± 0.11b	13.31 ± 0.40c	6.80 ± 0.05d	11.24 ± 0.01e
Artepillin C	0.00 ± 0.00a	13.25 ± 0.39b	41.82 ± 0.42c	4.03 ± 0.03d	6.65 ± 0.08e

The data is presented as the mean ± standard deviation of concentrations (mg/g) of three samples. Samples of green propolis were compared among themselves. The symbols correspond to statistical analysis. For each of the markers different symbols indicate differences among the samples (*P* < 0.05), while the similar symbols indicate no statistical difference among the samples. Analyzed by *one-way* ANOVA test followed by Bonferroni's multiple comparisons.

**Table 2:** The effect of oral treatment with propolis extracts from *Apis mellifera* in mice with granulomatous inflammation on the hematological parameters.

	Control	E1A	E1B	E10	E11	E12
Erythrocytes (×10^6^/*μ*L)	10.0 ± 0.3	10.2 ± 0.4	10.2 ± 0.64	9.5 ± 0.3	9.8 ± 0.2	9.8 ± 0.1
Hemoglobin (g/dL)	15.0 ± 0.2	15.2 ± 0.8	15.5 ± 0.5	14.4 ± 0.8	14.5 ± 0.5	14.8 ± 0.2
Hematocrit (%)	50.8 ± 0.5	51.7 ± 1.1	52.8 ± 0.6	48.3 ± 2.2	49.4 ± 1.3	50.2 ± 1.1
MCV (fL)^a^	50.7 ± 1.1	50.8 ± 0.9	50.4 ± 1.24	51 ± 1.1	50.3 ± 1.8	51.2 ± 0.7
MCH (pg)^b^	14.9 ± 0.3	14.9 ± 0.35	14.7 ± 0.66	15.2 ± 0.4	14.7 ± 0.8	15.2 ± 0.1
MCHC (g/dL)^c^	29.5 ± 0.2	29.4 ± 1.0	29.2 ± 0.8	29.8 ± 0.3	29.3 ± 0.6	29.6 ± 0.4
RDW-CV (%)^d^	17.1 ± 0.6	16.5 ± 0.6	17.1 ± 2.4	17.4 ± 0.5	17.0 ± 0.8	16.9 ± 0.1
Leukocytes (×10^3^/*μ*L)	9.3 ± 0.8	8.8 ± 0.97	8.7 ± 0.7	12.7 ± 1.0*	13.2 ± 1.0*	10.2 ± 1.0
Neutrophils (×10^3^/*μ*L)	0.8 ± 0.0	0.8 ± 0.1	0.9 ± 0.1	1.0 ± 0.0	1.2 ± 0.1	1.1 ± 0.1
Lymphocytes (×10^3^/*μ*L)	7.7 ± 0.7	6.9 ± 0.86	6.75 ± 0.35	9.6 ± 1.2	9.1 ± 2.12	8.3 ± 0.8
Platelets (×10^3^/*μ*L)	1286.0 ± 218.7	1164.0 ± 105.3	1426.0 ± 430.2	1328.0 ± 212.8	361.5 ± 289.2*	1318.0 ± 121

The results are presented as the mean ± standard deviation, ^a^MCV: mean corpuscular volume, ^b^MCH: mean corpuscular hemoglobin, ^c^MCHC: mean corpuscular hemoglobin concentration, ^d^RDW-CV: red cell distribution width, coefficient of variation. *P < 0.05 when compared to the control group.

**Table 3:** The number of cells of the lymphoid organs of mice with granulomatous inflammation orally treated with propolis extracts from *Apis mellifera*.

	Control	E1A	E1B	E10	E11	E12
Bone marrow (×10^6^/mL)	15.7 ± 0.2	14.1 ± 0.9	7.9 ± 0.9*	9.1 ± 0.3*	8.2 ± 0.3*	19.0 ± 1.2*
Spleen (×10^7^/mL)	2.4 ± 0.2	1.6 ± 0.1*	1.6 ± 0.1*	1.6 ± 0.2*	2.4 ± 0.1	2.6 ± 0.1
Lymph node (×10^6^/mL)	2.1 ± 0.5	7.4 ± 0.7*	2.2 ± 0.9	4.4 ± 0.2*	5.9 ± 0.5*	1.6 ± 0.2

The data is presented as the mean ± E.P.M. *P < 0.05 of replicates when compared to the control group.
